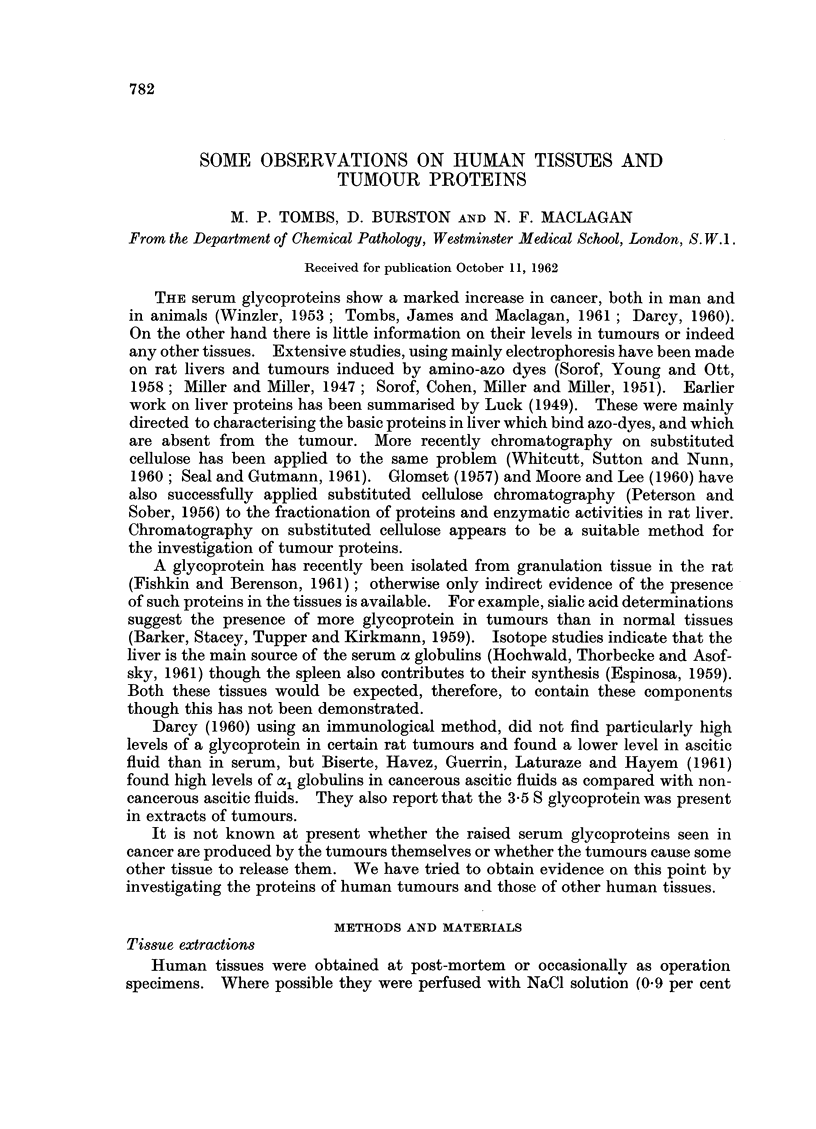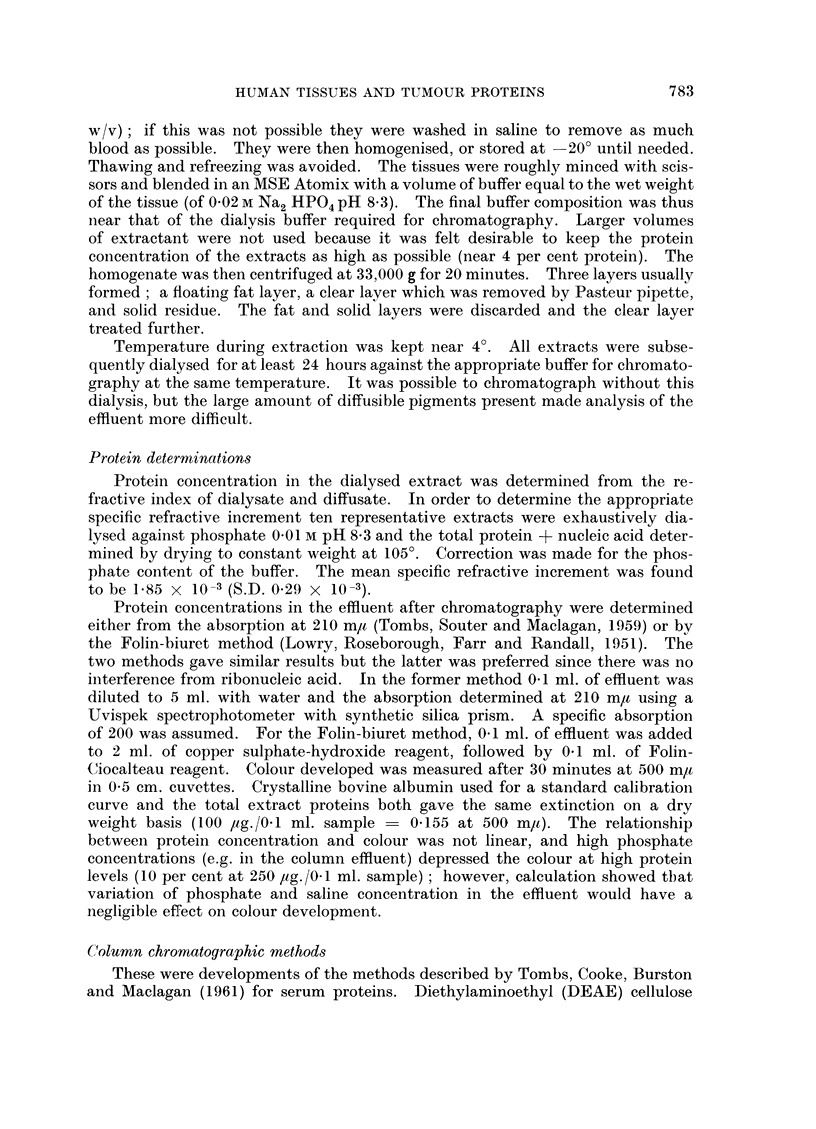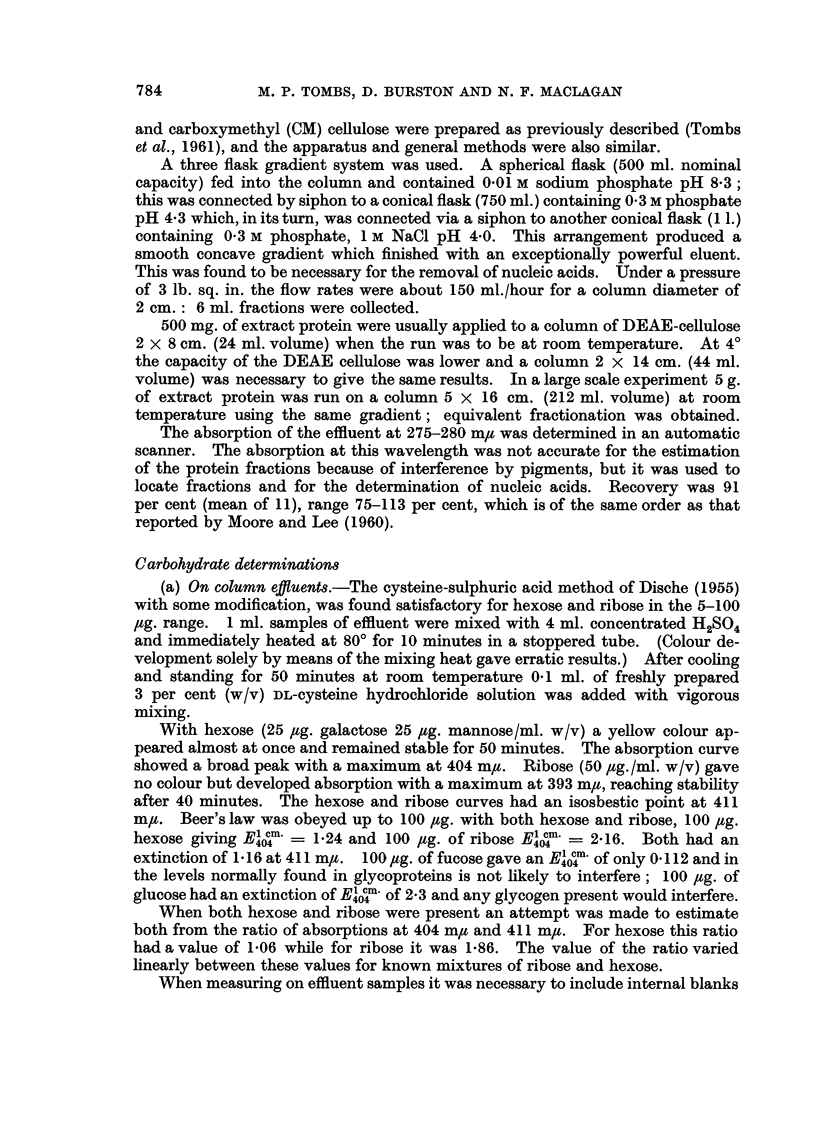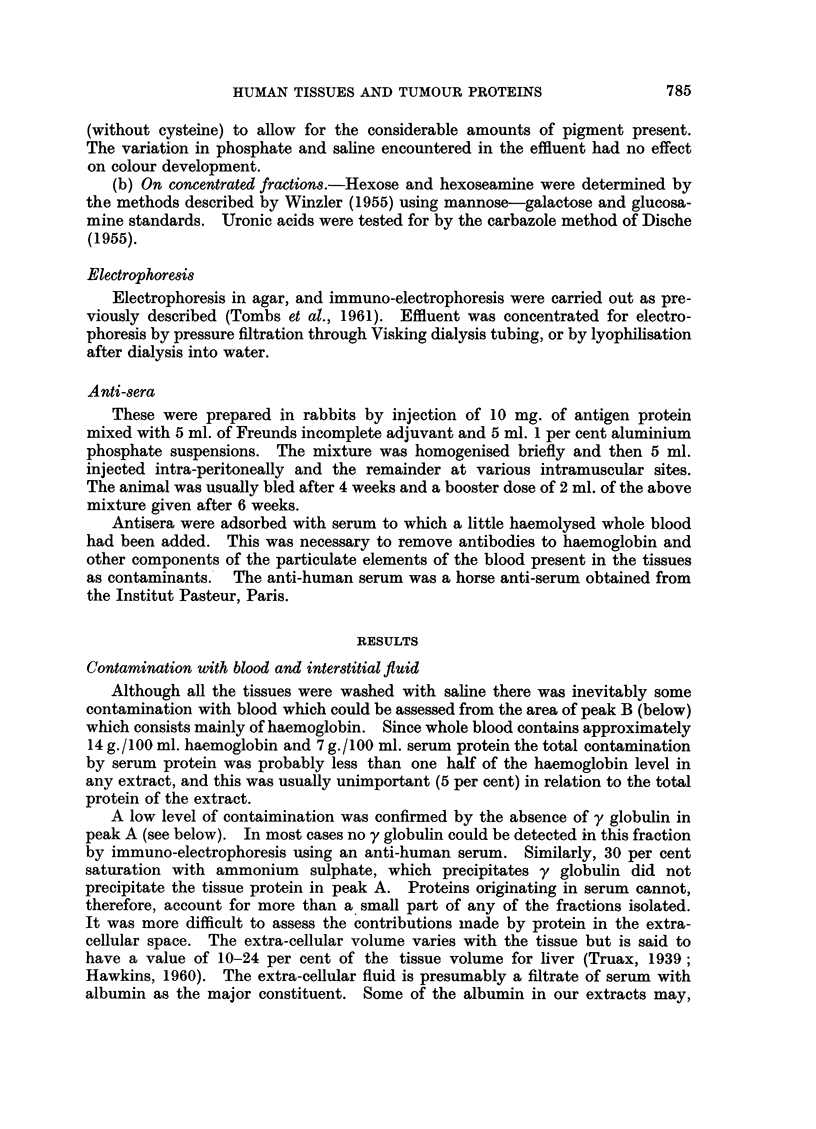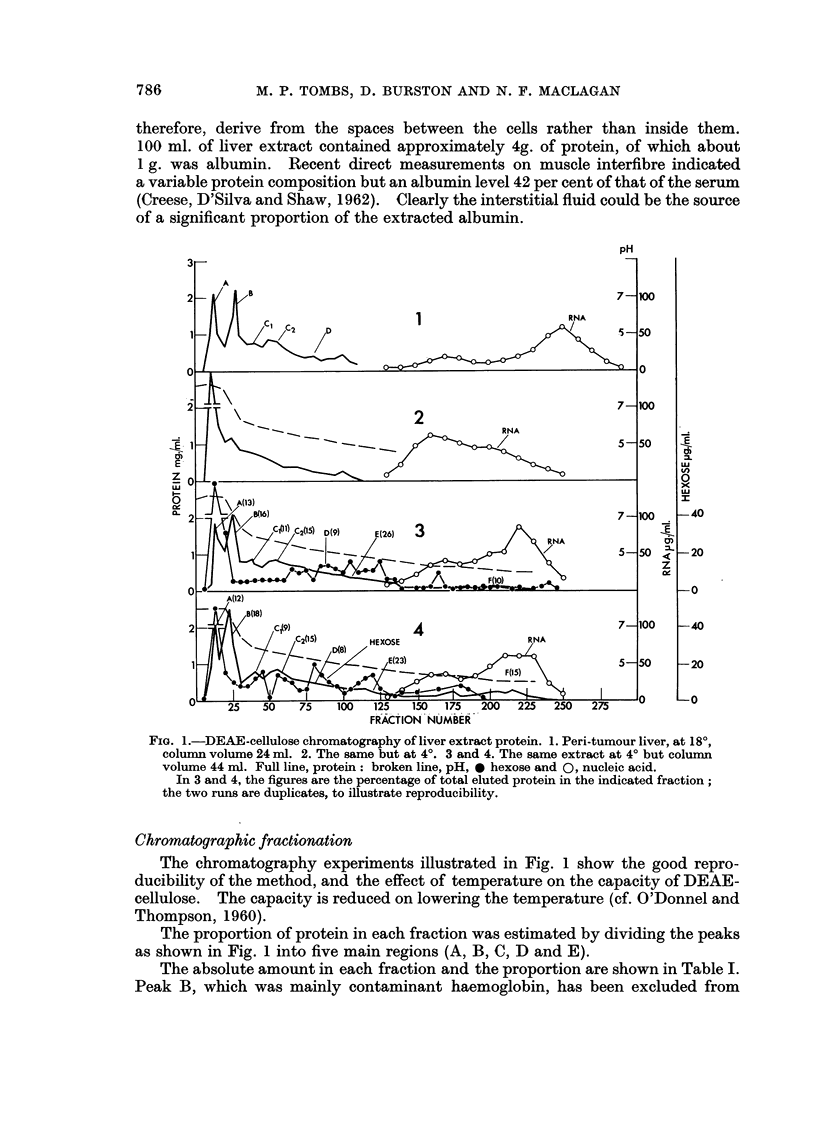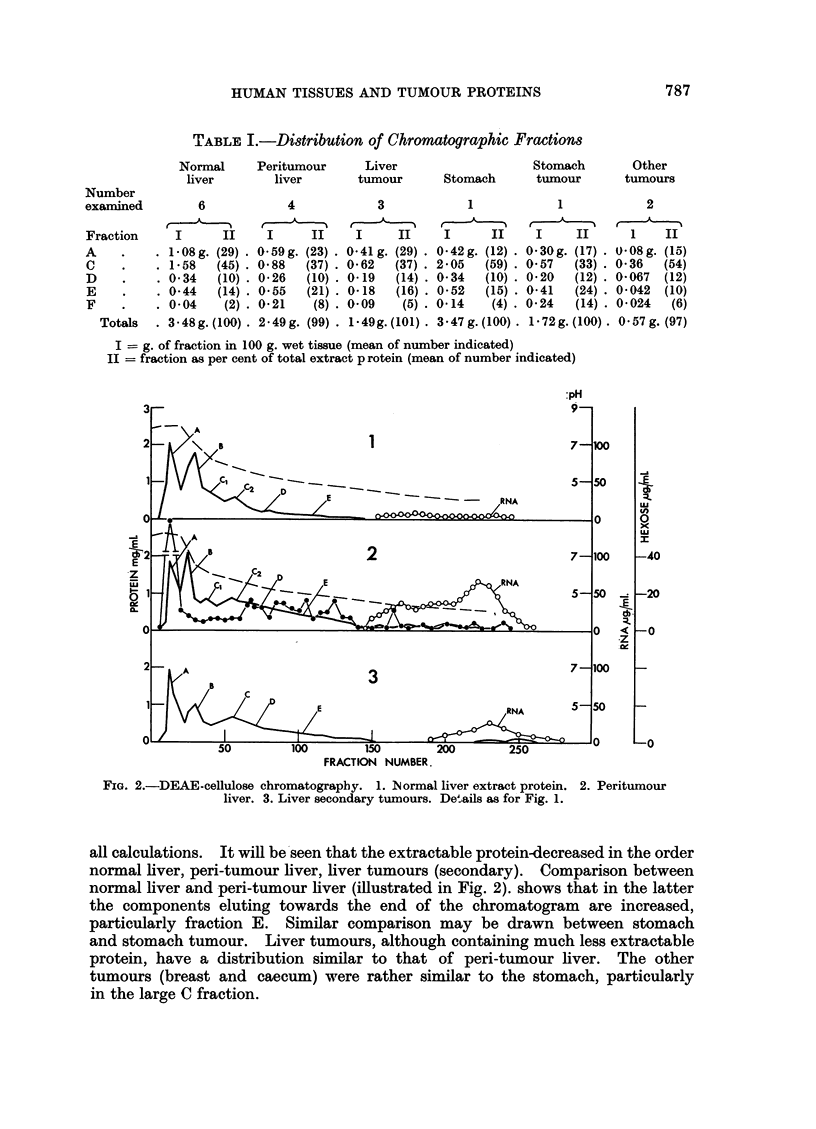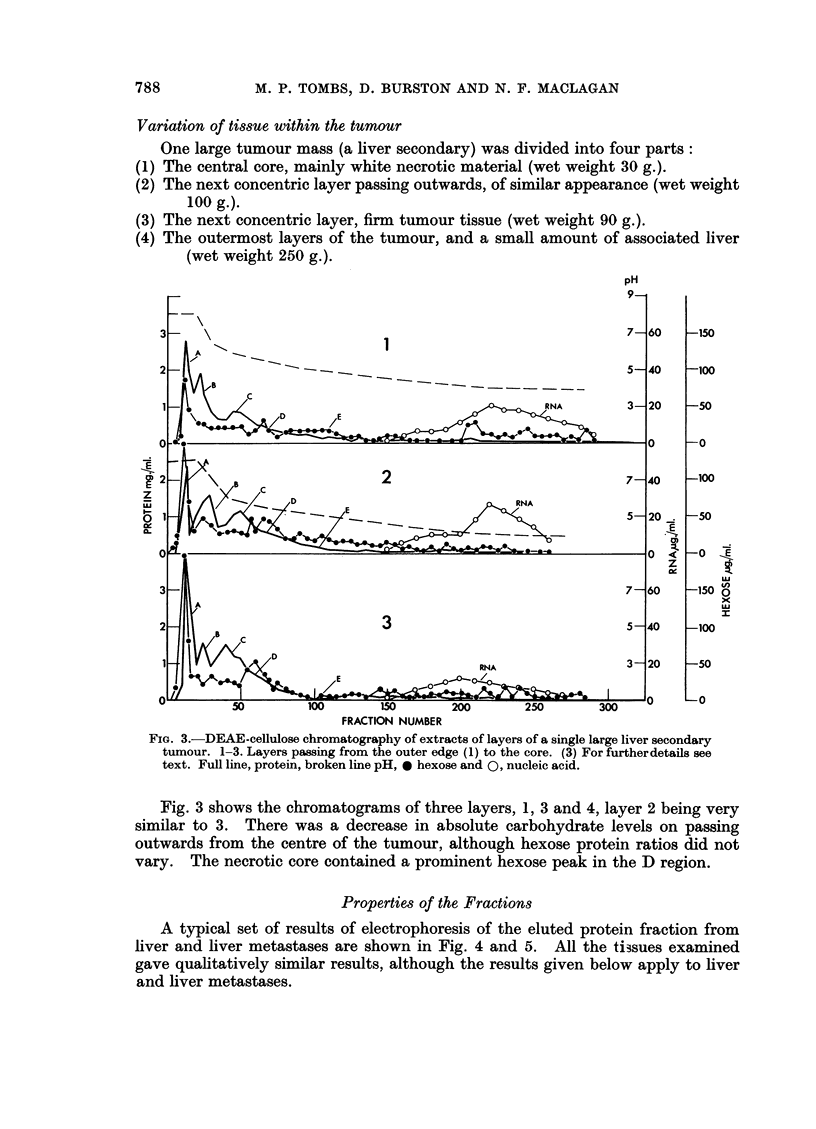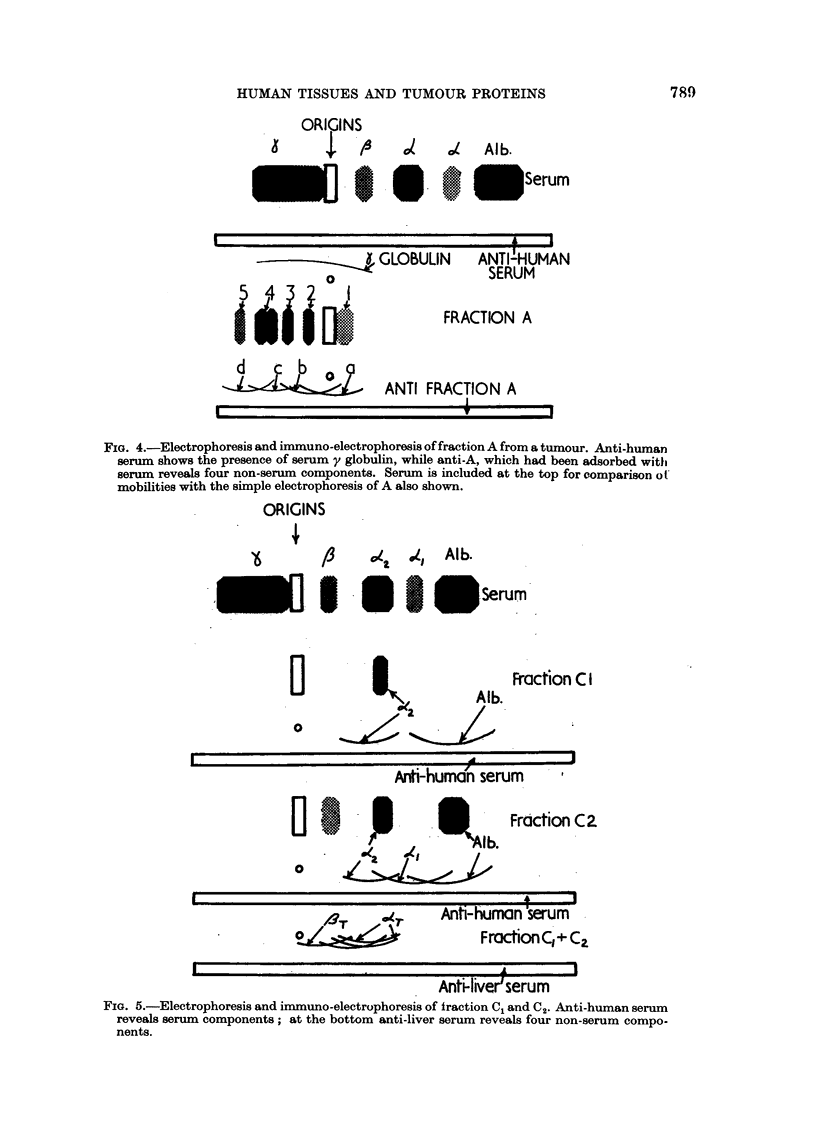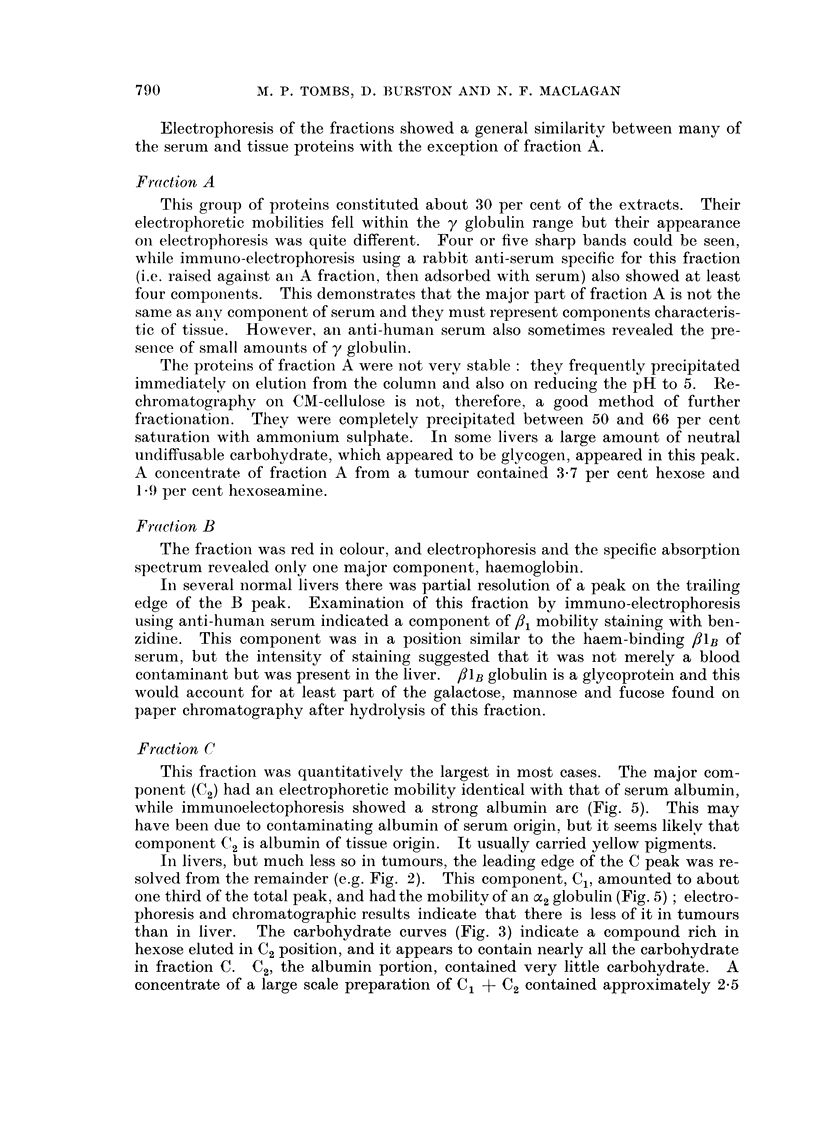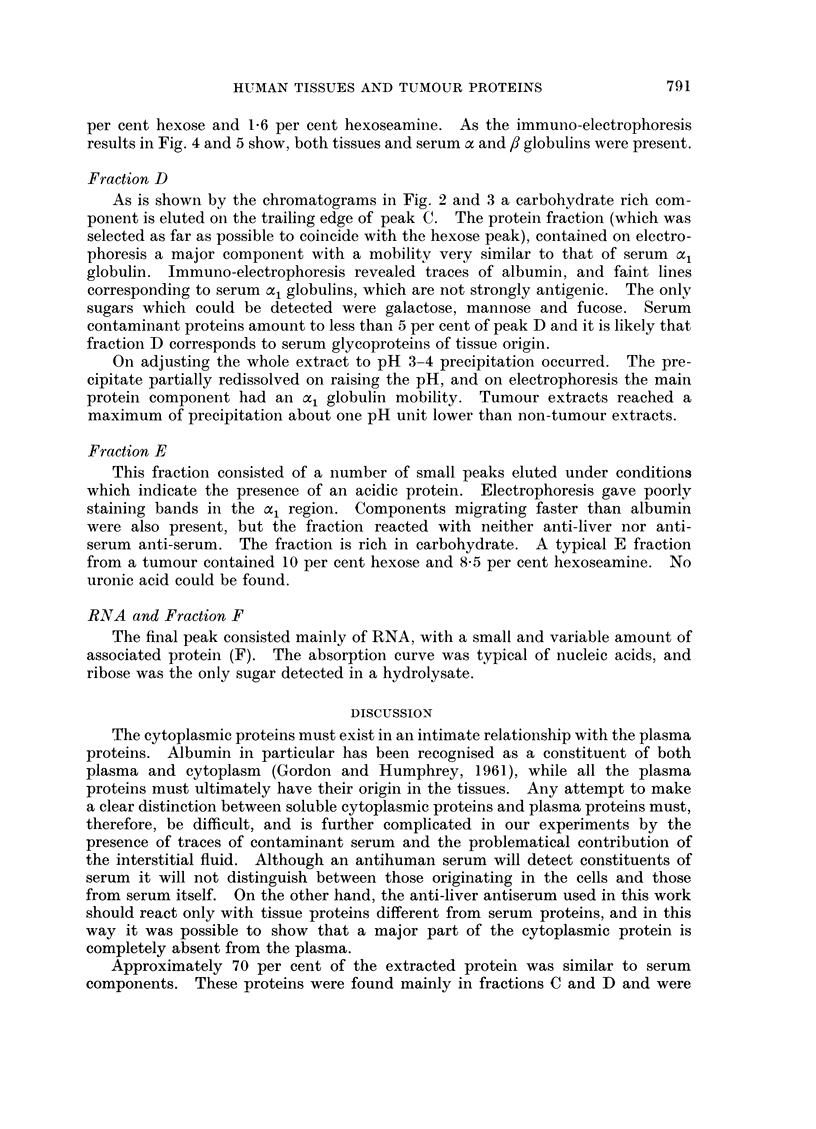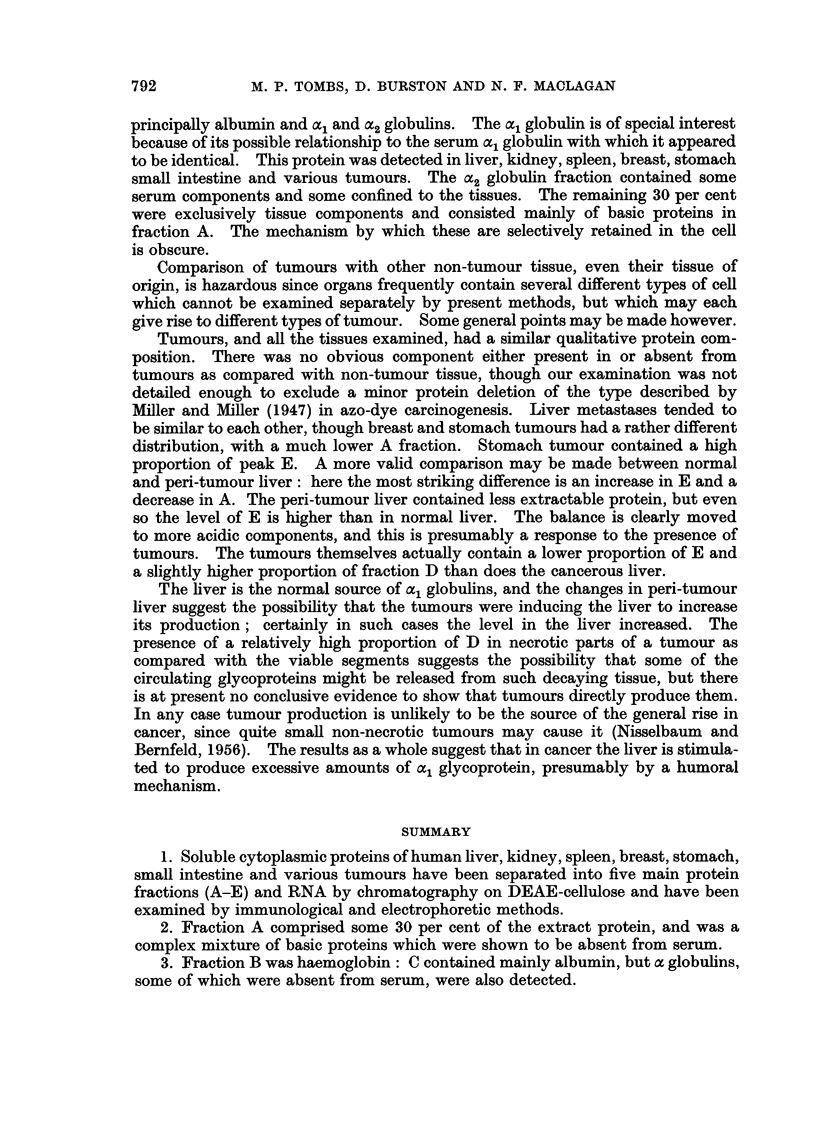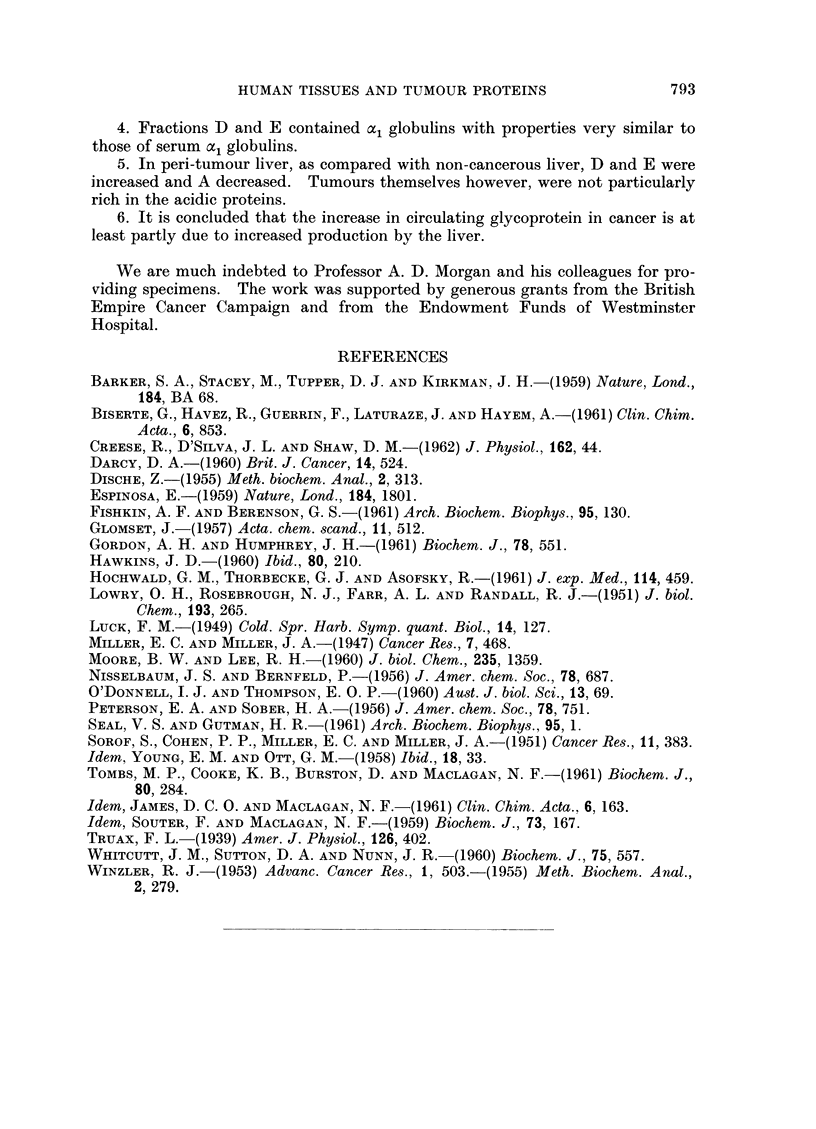# Some Observations on Human Tissues and Tumour Proteins

**DOI:** 10.1038/bjc.1962.91

**Published:** 1962-12

**Authors:** M. P. Tombs, D. Burston, N. F. Maclagan


### SOME OBSERVATIONS ON HUMAN TISSUES AND TUMOUR PROTEINS

M. P. TOMBS, D. BURSTON AND N. F. MACLAGAN

*From the Department of Chemical Pathology, Westminster Medical School, London, S.W.1.*

Received for publication October 11, 1962

THE serum glycoproteins show a marked increase in cancer, both in man and in animals (Winzler, 1953 ; Tombs, James and Maclagan, 1961 ; Darcy, 1960). On the other hand there is little information on their levels in tumours or indeed any other tissues. Extensive studies, using mainly electrophoresis have been made on rat livers and tumours induced by amino-azo dyes (Sorof, Young and Ott, 1958 ; Miller and Miller, 1947 ; Sorof, Cohen, Miller and Miller, 1951). Earlier work on liver proteins has been summarised by Luck (1949). These were mainly directed to characterising the basic proteins in liver which bind azo-dyes, and which are absent from the tumour. More recently chromatography on substituted cellulose has been applied to the same problem (Whitcutt, Sutton and Nunn, 1960 ; Seal and Gutmann, 1961). Glomset (1957) and Moore and Lee (1960) have also successfully applied substituted cellulose chromatography (Peterson and Sober, 1956) to the fractionation of proteins and enzymatic activities in rat liver. Chromatography on substituted cellulose appears to be a suitable method for the investigation of tumour proteins.

A glycoprotein has recently been isolated from granulation tissue in the rat (Fishkin and Berenson, 1961) ; otherwise only indirect evidence of the presence of such proteins in the tissues is available. For example, sialic acid determinations suggest the presence of more glycoprotein in tumours than in normal tissues (Barker, Stacey, Tupper and Kirkmann, 1959). Isotope studies indicate that the liver is the main source of the serum $\alpha$ globulins (Hochwald, Thorbecke and Asofsky, 1961) though the spleen also contributes to their synthesis (Espinosa, 1959). Both these tissues would be expected, therefore, to contain these components though this has not been demonstrated.

Darcy (1960) using an immunological method, did not find particularly high levels of a glycoprotein in certain rat tumours and found a lower level in ascitic fluid than in serum, but Biserte, Havez, Guerrin, Laturaze and Hayem (1961) found high levels of $\alpha_1$ globulins in cancerous ascitic fluids as compared with non-cancerous ascitic fluids. They also report that the 3·5 S glycoprotein was present in extracts of tumours.

It is not known at present whether the raised serum glycoproteins seen in cancer are produced by the tumours themselves or whether the tumours cause some other tissue to release them. We have tried to obtain evidence on this point by investigating the proteins of human tumours and those of other human tissues.

### METHODS AND MATERIALS

### *Tissue extractions*

Human tissues were obtained at post-mortem or occasionally as operation specimens. Where possible they were perfused with NaCl solution (0·9 per cent w/v) ; if this was not possible they were washed in saline to remove as much blood as possible. They were then homogenised, or stored at $-20°$ until needed. Thawing and refreezing was avoided. The tissues were roughly minced with scissors and blended in an MSE Atomix with a volume of buffer equal to the wet weight of the tissue (of 0·02 M $Na_2$ $HPO_4$ pH 8·3). The final buffer composition was thus near that of the dialysis buffer required for chromatography. Larger volumes of extractant were not used because it was felt desirable to keep the protein concentration of the extracts as high as possible (near 4 per cent protein). The homogenate was then centrifuged at 33,000 **g** for 20 minutes. Three layers usually formed ; a floating fat layer, a clear layer which was removed by Pasteur pipette, and solid residue. The fat and solid layers were discarded and the clear layer treated further.

Temperature during extraction was kept near 4°. All extracts were subsequently dialysed for at least 24 hours against the appropriate buffer for chromatography at the same temperature. It was possible to chromatograph without this dialysis, but the large amount of diffusible pigments present made analysis of the effluent more difficult.

*Protein determinations*

Protein concentration in the dialysed extract was determined from the refractive index of dialysate and diffusate. In order to determine the appropriate specific refractive increment ten representative extracts were exhaustively dialysed against phosphate 0·01 M pH 8·3 and the total protein + nucleic acid determined by drying to constant weight at 105°. Correction was made for the phosphate content of the buffer. The mean specific refractive increment was found to be $1{\cdot}85 \times 10^{-3}$ (S.D. $0{\cdot}29 \times 10^{-3}$).

Protein concentrations in the effluent after chromatography were determined either from the absorption at 210 m$\mu$ (Tombs, Souter and Maclagan, 1959) or by the Folin-biuret method (Lowry, Roseborough, Farr and Randall, 1951). The two methods gave similar results but the latter was preferred since there was no interference from ribonucleic acid. In the former method 0·1 ml. of effluent was diluted to 5 ml. with water and the absorption determined at 210 m$\mu$ using a Uvispek spectrophotometer with synthetic silica prism. A specific absorption of 200 was assumed. For the Folin-biuret method, 0·1 ml. of effluent was added to 2 ml. of copper sulphate-hydroxide reagent, followed by 0·1 ml. of Folin-Ciocalteau reagent. Colour developed was measured after 30 minutes at 500 m$\mu$ in 0·5 cm. cuvettes. Crystalline bovine albumin used for a standard calibration curve and the total extract proteins both gave the same extinction on a dry weight basis (100 $\mu$g./0·1 ml. sample = 0·155 at 500 m$\mu$). The relationship between protein concentration and colour was not linear, and high phosphate concentrations (e.g. in the column effluent) depressed the colour at high protein levels (10 per cent at 250 $\mu$g./0·1 ml. sample) ; however, calculation showed that variation of phosphate and saline concentration in the effluent would have a negligible effect on colour development.

*Column chromatographic methods*

These were developments of the methods described by Tombs, Cooke, Burston and Maclagan (1961) for serum proteins. Diethylaminoethyl (DEAE) cellulose and carboxymethyl (CM) cellulose were prepared as previously described (Tombs *et al.*, 1961), and the apparatus and general methods were also similar.

A three flask gradient system was used. A spherical flask (500 ml. nominal capacity) fed into the column and contained 0·01 M sodium phosphate pH 8·3; this was connected by siphon to a conical flask (750 ml.) containing 0·3 M phospbate pH 4·3 which, in its turn, was connected via a siphon to another conical flask (1 l.) containing 0·3 M phosphate, 1 M NaCl pH 4·0. This arrangement produced a smooth concave gradient which finished with an exceptionally powerful eluent. This was found to be necessary for the removal of nucleic acids. Under a pressure of 3 lb. sq. in. the flow rates were about 150 ml./hour for a column diameter of 2 cm.: 6 ml. fractions were collected.

500 mg. of extract protein were usually applied to a column of DEAE-cellulose 2 × 8 cm. (24 ml. volume) when the run was to be at room temperature. At 4° the capacity of the DEAE cellulose was lower and a column 2 × 14 cm. (44 ml. volume) was necessary to give the same results. In a large scale experiment 5 g. of extract protein was run on a column 5 × 16 cm. (212 ml. volume) at room temperature using the same gradient; equivalent fractionation was obtained.

The absorption of the effluent at 275–280 mμ was determined in an automatic scanner. The absorption at this wavelength was not accurate for the estimation of the protein fractions because of interference by pigments, but it was used to locate fractions and for the determination of nucleic acids. Recovery was 91 per cent (mean of 11), range 75–113 per cent, which is of the same order as that reported by Moore and Lee (1960).

*Carbohydrate determinations*

(a) *On column effluents.*—The cysteine-sulphuric acid method of Dische (1955) with some modification, was found satisfactory for hexose and ribose in the 5–100 μg. range. 1 ml. samples of effluent were mixed with 4 ml. concentrated $H_2SO_4$ and immediately heated at 80° for 10 minutes in a stoppered tube. (Colour development solely by means of the mixing heat gave erratic results.) After cooling and standing for 50 minutes at room temperature 0·1 ml. of freshly prepared 3 per cent (w/v) DL-cysteine hydrochloride solution was added with vigorous mixing.

With hexose (25 μg. galactose 25 μg. mannose/ml. w/v) a yellow colour appeared almost at once and remained stable for 50 minutes. The absorption curve showed a broad peak with a maximum at 404 mμ. Ribose (50 μg./ml. w/v) gave no colour but developed absorption with a maximum at 393 mμ, reaching stability after 40 minutes. The hexose and ribose curves had an isosbestic point at 411 mμ. Beer's law was obeyed up to 100 μg. with both hexose and ribose, 100 μg. hexose giving $E_{404}^{1\,cm.} = 1{\cdot}24$ and 100 μg. of ribose $E_{404}^{1\,cm.} = 2{\cdot}16$. Both had an extinction of 1·16 at 411 mμ. 100 μg. of fucose gave an $E_{404}^{1\,cm.}$ of only 0·112 and in the levels normally found in glycoproteins is not likely to interfere; 100 μg. of glucose had an extinction of $E_{404}^{1\,cm.}$ of 2·3 and any glycogen present would interfere.

When both hexose and ribose were present an attempt was made to estimate both from the ratio of absorptions at 404 mμ and 411 mμ. For hexose this ratio had a value of 1·06 while for ribose it was 1·86. The value of the ratio varied linearly between these values for known mixtures of ribose and hexose.

When measuring on effluent samples it was necessary to include internal blanks

(without cysteine) to allow for the considerable amounts of pigment present. The variation in phosphate and saline encountered in the effluent had no effect on colour development.

(b) *On concentrated fractions.*—Hexose and hexoseamine were determined by the methods described by Winzler (1955) using mannose—galactose and glucosamine standards. Uronic acids were tested for by the carbazole method of Dische (1955).

*Electrophoresis*

Electrophoresis in agar, and immuno-electrophoresis were carried out as previously described (Tombs *et al.*, 1961). Effluent was concentrated for electrophoresis by pressure filtration through Visking dialysis tubing, or by lyophilisation after dialysis into water.

*Anti-sera*

These were prepared in rabbits by injection of 10 mg. of antigen protein mixed with 5 ml. of Freunds incomplete adjuvant and 5 ml. 1 per cent aluminium phosphate suspensions. The mixture was homogenised briefly and then 5 ml. injected intra-peritoneally and the remainder at various intramuscular sites. The animal was usually bled after 4 weeks and a booster dose of 2 ml. of the above mixture given after 6 weeks.

Antisera were adsorbed with serum to which a little haemolysed whole blood had been added. This was necessary to remove antibodies to haemoglobin and other components of the particulate elements of the blood present in the tissues as contaminants. The anti-human serum was a horse anti-serum obtained from the Institut Pasteur, Paris.

### RESULTS

*Contamination with blood and interstitial fluid*

Although all the tissues were washed with saline there was inevitably some contamination with blood which could be assessed from the area of peak B (below) which consists mainly of haemoglobin. Since whole blood contains approximately 14 g./100 ml. haemoglobin and 7 g./100 ml. serum protein the total contamination by serum protein was probably less than one half of the haemoglobin level in any extract, and this was usually unimportant (5 per cent) in relation to the total protein of the extract.

A low level of contaimination was confirmed by the absence of $\gamma$ globulin in peak A (see below). In most cases no $\gamma$ globulin could be detected in this fraction by immuno-electrophoresis using an anti-human serum. Similarly, 30 per cent saturation with ammonium sulphate, which precipitates $\gamma$ globulin did not precipitate the tissue protein in peak A. Proteins originating in serum cannot, therefore, account for more than a small part of any of the fractions isolated. It was more difficult to assess the contributions made by protein in the extra-cellular space. The extra-cellular volume varies with the tissue but is said to have a value of 10–24 per cent of the tissue volume for liver (Truax, 1939; Hawkins, 1960). The extra-cellular fluid is presumably a filtrate of serum with albumin as the major constituent. Some of the albumin in our extracts may, therefore, derive from the spaces between the cells rather than inside them. 100 ml. of liver extract contained approximately 4g. of protein, of which about 1 g. was albumin. Recent direct measurements on muscle interfibre indicated a variable protein composition but an albumin level 42 per cent of that of the serum (Creese, D'Silva and Shaw, 1962). Clearly the interstitial fluid could be the source of a significant proportion of the extracted albumin.

FIG. 1.—DEAE-cellulose chromatography of liver extract protein. 1. Peri-tumour liver, at 18°, column volume 24 ml. 2. The same but at 4°. 3 and 4. The same extract at 4° but column volume 44 ml. Full line, protein : broken line, pH, ● hexose and ○, nucleic acid.

In 3 and 4, the figures are the percentage of total eluted protein in the indicated fraction ; the two runs are duplicates, to illustrate reproducibility.

*Chromatographic fractionation*

The chromatography experiments illustrated in Fig. 1 show the good reproducibility of the method, and the effect of temperature on the capacity of DEAE-cellulose. The capacity is reduced on lowering the temperature (cf. O'Donnel and Thompson, 1960).

The proportion of protein in each fraction was estimated by dividing the peaks as shown in Fig. 1 into five main regions (A, B, C, D and E).

The absolute amount in each fraction and the proportion are shown in Table I. Peak B, which was mainly contaminant haemoglobin, has been excluded from

TABLE I.—*Distribution of Chromatographic Fractions*

| | Normal liver | | Peritumour liver | | Liver tumour | | Stomach | | Stomach tumour | | Other tumours | |
|---|---|---|---|---|---|---|---|---|---|---|---|---|
| Number examined | 6 | | 4 | | 3 | | 1 | | 1 | | 2 | |
| Fraction | I | II | I | II | I | II | I | II | I | II | I | II |
| A | 1·08 g. | (29) | 0·59 g. | (23) | 0·41 g. | (29) | 0·42 g. | (12) | 0·30 g. | (17) | 0·08 g. | (15) |
| C | 1·58 | (45) | 0·88 | (37) | 0·62 | (37) | 2·05 | (59) | 0·57 | (33) | 0·36 | (54) |
| D | 0·34 | (10) | 0·26 | (10) | 0·19 | (14) | 0·34 | (10) | 0·20 | (12) | 0·067 | (12) |
| E | 0·44 | (14) | 0·55 | (21) | 0·18 | (16) | 0·52 | (15) | 0·41 | (24) | 0·042 | (10) |
| F | 0·04 | (2) | 0·21 | (8) | 0·09 | (5) | 0·14 | (4) | 0·24 | (14) | 0·024 | (6) |
| Totals | 3·48 g. | (100) | 2·49 g. | (99) | 1·49 g. | (101) | 3·47 g. | (100) | 1·72 g. | (100) | 0·57 g. | (97) |

I = g. of fraction in 100 g. wet tissue (mean of number indicated)
II = fraction as per cent of total extract protein (mean of number indicated)

FIG. 2.—DEAE-cellulose chromatography. 1. Normal liver extract protein. 2. Peritumour liver. 3. Liver secondary tumours. Details as for Fig. 1.

all calculations. It will be seen that the extractable protein decreased in the order normal liver, peri-tumour liver, liver tumours (secondary). Comparison between normal liver and peri-tumour liver (illustrated in Fig. 2). shows that in the latter the components eluting towards the end of the chromatogram are increased, particularly fraction E. Similar comparison may be drawn between stomach and stomach tumour. Liver tumours, although containing much less extractable protein, have a distribution similar to that of peri-tumour liver. The other tumours (breast and caecum) were rather similar to the stomach, particularly in the large C fraction.

*Variation of tissue within the tumour*

One large tumour mass (a liver secondary) was divided into four parts :

(1) The central core, mainly white necrotic material (wet weight 30 g.).

(2) The next concentric layer passing outwards, of similar appearance (wet weight 100 g.).

(3) The next concentric layer, firm tumour tissue (wet weight 90 g.).

(4) The outermost layers of the tumour, and a small amount of associated liver (wet weight 250 g.).

FIG. 3.—DEAE-cellulose chromatography of extracts of layers of a single large liver secondary tumour. 1–3. Layers passing from the outer edge (1) to the core. (3) For further details see text. Full line, protein, broken line pH, ● hexose and ○, nucleic acid.

Fig. 3 shows the chromatograms of three layers, 1, 3 and 4, layer 2 being very similar to 3. There was a decrease in absolute carbohydrate levels on passing outwards from the centre of the tumour, although hexose protein ratios did not vary. The necrotic core contained a prominent hexose peak in the D region.

### *Properties of the Fractions*

A typical set of results of electrophoresis of the eluted protein fraction from liver and liver metastases are shown in Fig. 4 and 5. All the tissues examined gave qualitatively similar results, although the results given below apply to liver and liver metastases.

FIG. 4.—Electrophoresis and immuno-electrophoresis of fraction A from a tumour. Anti-human serum shows the presence of serum $\gamma$ globulin, while anti-A, which had been adsorbed with serum reveals four non-serum components. Serum is included at the top for comparison of mobilities with the simple electrophoresis of A also shown.

FIG. 5.—Electrophoresis and immuno-electrophoresis of fraction $C_1$ and $C_2$. Anti-human serum reveals serum components ; at the bottom anti-liver serum reveals four non-serum components.

Electrophoresis of the fractions showed a general similarity between many of the serum and tissue proteins with the exception of fraction A.

*Fraction A*

This group of proteins constituted about 30 per cent of the extracts. Their electrophoretic mobilities fell within the $\gamma$ globulin range but their appearance on electrophoresis was quite different. Four or five sharp bands could be seen, while immuno-electrophoresis using a rabbit anti-serum specific for this fraction (i.e. raised against an A fraction, then adsorbed with serum) also showed at least four components. This demonstrates that the major part of fraction A is not the same as any component of serum and they must represent components characteristic of tissue. However, an anti-human serum also sometimes revealed the presence of small amounts of $\gamma$ globulin.

The proteins of fraction A were not very stable : they frequently precipitated immediately on elution from the column and also on reducing the pH to 5. Rechromatography on CM-cellulose is not, therefore, a good method of further fractionation. They were completely precipitated between 50 and 66 per cent saturation with ammonium sulphate. In some livers a large amount of neutral undiffusable carbohydrate, which appeared to be glycogen, appeared in this peak. A concentrate of fraction A from a tumour contained 3·7 per cent hexose and 1·9 per cent hexoseamine.

*Fraction B*

The fraction was red in colour, and electrophoresis and the specific absorption spectrum revealed only one major component, haemoglobin.

In several normal livers there was partial resolution of a peak on the trailing edge of the B peak. Examination of this fraction by immuno-electrophoresis using anti-human serum indicated a component of $\beta_1$ mobility staining with benzidine. This component was in a position similar to the haem-binding $\beta 1_B$ of serum, but the intensity of staining suggested that it was not merely a blood contaminant but was present in the liver. $\beta 1_B$ globulin is a glycoprotein and this would account for at least part of the galactose, mannose and fucose found on paper chromatography after hydrolysis of this fraction.

*Fraction C*

This fraction was quantitatively the largest in most cases. The major component ($C_2$) had an electrophoretic mobility identical with that of serum albumin, while immunoelectophoresis showed a strong albumin arc (Fig. 5). This may have been due to contaminating albumin of serum origin, but it seems likely that component $C_2$ is albumin of tissue origin. It usually carried yellow pigments.

In livers, but much less so in tumours, the leading edge of the C peak was resolved from the remainder (e.g. Fig. 2). This component, $C_1$, amounted to about one third of the total peak, and had the mobility of an $\alpha_2$ globulin (Fig. 5) ; electrophoresis and chromatographic results indicate that there is less of it in tumours than in liver. The carbohydrate curves (Fig. 3) indicate a compound rich in hexose eluted in $C_2$ position, and it appears to contain nearly all the carbohydrate in fraction C. $C_2$, the albumin portion, contained very little carbohydrate. A concentrate of a large scale preparation of $C_1 + C_2$ contained approximately 2·5

per cent hexose and 1·6 per cent hexoseamine. As the immuno-electrophoresis results in Fig. 4 and 5 show, both tissues and serum $\alpha$ and $\beta$ globulins were present.

*Fraction D*

As is shown by the chromatograms in Fig. 2 and 3 a carbohydrate rich component is eluted on the trailing edge of peak C. The protein fraction (which was selected as far as possible to coincide with the hexose peak), contained on electrophoresis a major component with a mobility very similar to that of serum $\alpha_1$ globulin. Immuno-electrophoresis revealed traces of albumin, and faint lines corresponding to serum $\alpha_1$ globulins, which are not strongly antigenic. The only sugars which could be detected were galactose, mannose and fucose. Serum contaminant proteins amount to less than 5 per cent of peak D and it is likely that fraction D corresponds to serum glycoproteins of tissue origin.

On adjusting the whole extract to pH 3–4 precipitation occurred. The precipitate partially redissolved on raising the pH, and on electrophoresis the main protein component had an $\alpha_1$ globulin mobility. Tumour extracts reached a maximum of precipitation about one pH unit lower than non-tumour extracts.

*Fraction E*

This fraction consisted of a number of small peaks eluted under conditions which indicate the presence of an acidic protein. Electrophoresis gave poorly staining bands in the $\alpha_1$ region. Components migrating faster than albumin were also present, but the fraction reacted with neither anti-liver nor anti-serum anti-serum. The fraction is rich in carbohydrate. A typical E fraction from a tumour contained 10 per cent hexose and 8·5 per cent hexoseamine. No uronic acid could be found.

*RNA and Fraction F*

The final peak consisted mainly of RNA, with a small and variable amount of associated protein (F). The absorption curve was typical of nucleic acids, and ribose was the only sugar detected in a hydrolysate.

### DISCUSSION

The cytoplasmic proteins must exist in an intimate relationship with the plasma proteins. Albumin in particular has been recognised as a constituent of both plasma and cytoplasm (Gordon and Humphrey, 1961), while all the plasma proteins must ultimately have their origin in the tissues. Any attempt to make a clear distinction between soluble cytoplasmic proteins and plasma proteins must, therefore, be difficult, and is further complicated in our experiments by the presence of traces of contaminant serum and the problematical contribution of the interstitial fluid. Although an antihuman serum will detect constituents of serum it will not distinguish between those originating in the cells and those from serum itself. On the other hand, the anti-liver antiserum used in this work should react only with tissue proteins different from serum proteins, and in this way it was possible to show that a major part of the cytoplasmic protein is completely absent from the plasma.

Approximately 70 per cent of the extracted protein was similar to serum components. These proteins were found mainly in fractions C and D and were principally albumin and $\alpha_1$ and $\alpha_2$ globulins. The $\alpha_1$ globulin is of special interest because of its possible relationship to the serum $\alpha_1$ globulin with which it appeared to be identical. This protein was detected in liver, kidney, spleen, breast, stomach small intestine and various tumours. The $\alpha_2$ globulin fraction contained some serum components and some confined to the tissues. The remaining 30 per cent were exclusively tissue components and consisted mainly of basic proteins in fraction A. The mechanism by which these are selectively retained in the cell is obscure.

Comparison of tumours with other non-tumour tissue, even their tissue of origin, is hazardous since organs frequently contain several different types of cell which cannot be examined separately by present methods, but which may each give rise to different types of tumour. Some general points may be made however.

Tumours, and all the tissues examined, had a similar qualitative protein composition. There was no obvious component either present in or absent from tumours as compared with non-tumour tissue, though our examination was not detailed enough to exclude a minor protein deletion of the type described by Miller and Miller (1947) in azo-dye carcinogenesis. Liver metastases tended to be similar to each other, though breast and stomach tumours had a rather different distribution, with a much lower A fraction. Stomach tumour contained a high proportion of peak E. A more valid comparison may be made between normal and peri-tumour liver : here the most striking difference is an increase in E and a decrease in A. The peri-tumour liver contained less extractable protein, but even so the level of E is higher than in normal liver. The balance is clearly moved to more acidic components, and this is presumably a response to the presence of tumours. The tumours themselves actually contain a lower proportion of E and a slightly higher proportion of fraction D than does the cancerous liver.

The liver is the normal source of $\alpha_1$ globulins, and the changes in peri-tumour liver suggest the possibility that the tumours were inducing the liver to increase its production ; certainly in such cases the level in the liver increased. The presence of a relatively high proportion of D in necrotic parts of a tumour as compared with the viable segments suggests the possibility that some of the circulating glycoproteins might be released from such decaying tissue, but there is at present no conclusive evidence to show that tumours directly produce them. In any case tumour production is unlikely to be the source of the general rise in cancer, since quite small non-necrotic tumours may cause it (Nisselbaum and Bernfeld, 1956). The results as a whole suggest that in cancer the liver is stimulated to produce excessive amounts of $\alpha_1$ glycoprotein, presumably by a humoral mechanism.

### SUMMARY

1. Soluble cytoplasmic proteins of human liver, kidney, spleen, breast, stomach, small intestine and various tumours have been separated into five main protein fractions (A–E) and RNA by chromatography on DEAE-cellulose and have been examined by immunological and electrophoretic methods.

2. Fraction A comprised some 30 per cent of the extract protein, and was a complex mixture of basic proteins which were shown to be absent from serum.

3. Fraction B was haemoglobin : C contained mainly albumin, but $\alpha$ globulins, some of which were absent from serum, were also detected.

4. Fractions D and E contained $\alpha_1$ globulins with properties very similar to those of serum $\alpha_1$ globulins.

5. In peri-tumour liver, as compared with non-cancerous liver, D and E were increased and A decreased. Tumours themselves however, were not particularly rich in the acidic proteins.

6. It is concluded that the increase in circulating glycoprotein in cancer is at least partly due to increased production by the liver.

We are much indebted to Professor A. D. Morgan and his colleagues for providing specimens. The work was supported by generous grants from the British Empire Cancer Campaign and from the Endowment Funds of Westminster Hospital.

### REFERENCES

BARKER, S. A., STACEY, M., TUPPER, D. J. AND KIRKMAN, J. H.—(1959) *Nature, Lond.*, **184**, BA 68.
BISERTE, G., HAVEZ, R., GUERRIN, F., LATURAZE, J. AND HAYEM, A.—(1961) *Clin. Chim. Acta.*, **6**, 853.
CREESE, R., D'SILVA, J. L. AND SHAW, D. M.—(1962) *J. Physiol.*, **162**, 44.
DARCY, D. A.—(1960) *Brit. J. Cancer*, **14**, 524.
DISCHE, Z.—(1955) *Meth. biochem. Anal.*, **2**, 313.
ESPINOSA, E.—(1959) *Nature, Lond.*, **184**, 1801.
FISHKIN, A. F. AND BERENSON, G. S.—(1961) *Arch. Biochem. Biophys.*, **95**, 130.
GLOMSET, J.—(1957) *Acta. chem. scand.*, **11**, 512.
GORDON, A. H. AND HUMPHREY, J. H.—(1961) *Biochem. J.*, **78**, 551.
HAWKINS, J. D.—(1960) *Ibid.*, **80**, 210.
HOCHWALD, G. M., THORBECKE, G. J. AND ASOFSKY, R.—(1961) *J. exp. Med.*, **114**, 459.
LOWRY, O. H., ROSEBROUGH, N. J., FARR, A. L. AND RANDALL, R. J.—(1951) *J. biol. Chem.*, **193**, 265.
LUCK, F. M.—(1949) *Cold. Spr. Harb. Symp. quant. Biol.*, **14**, 127.
MILLER, E. C. AND MILLER, J. A.—(1947) *Cancer Res.*, **7**, 468.
MOORE, B. W. AND LEE, R. H.—(1960) *J. biol. Chem.*, **235**, 1359.
NISSELBAUM, J. S. AND BERNFELD, P.—(1956) *J. Amer. chem. Soc.*, **78**, 687.
O'DONNELL, I. J. AND THOMPSON, E. O. P.—(1960) *Aust. J. biol. Sci.*, **13**, 69.
PETERSON, E. A. AND SOBER, H. A.—(1956) *J. Amer. chem. Soc.*, **78**, 751.
SEAL, V. S. AND GUTMAN, H. R.—(1961) *Arch. Biochem. Biophys.*, **95**, 1.
SOROF, S., COHEN, P. P., MILLER, E. C. AND MILLER, J. A.—(1951) *Cancer Res.*, **11**, 383.
*Idem*, YOUNG, E. M. AND OTT, G. M.—(1958) *Ibid.*, **18**, 33.
TOMBS, M. P., COOKE, K. B., BURSTON, D. AND MACLAGAN, N. F.—(1961) *Biochem. J.*, **80**, 284.
*Idem*, JAMES, D. C. O. AND MACLAGAN, N. F.—(1961) *Clin. Chim. Acta.*, **6**, 163.
*Idem*, SOUTER, F. AND MACLAGAN, N. F.—(1959) *Biochem. J.*, **73**, 167.
TRUAX, F. L.—(1939) *Amer. J. Physiol.*, **126**, 402.
WHITCUTT, J. M., SUTTON, D. A. AND NUNN, J. R.—(1960) *Biochem. J.*, **75**, 557.
WINZLER, R. J.—(1953) *Advanc. Cancer Res.*, **1**, 503.—(1955) *Meth. Biochem. Anal.*, **2**, 279.